# Clarifying differences between review designs and methods

**DOI:** 10.1186/2046-4053-1-28

**Published:** 2012-06-09

**Authors:** David Gough, James Thomas, Sandy Oliver

**Affiliations:** 1 EPPI-Centre, Social Science Research Unit, Institute of Education, University of London, 20 Bedford Way, London, WC1H 0AL, UK

**Keywords:** Aggregation configuration, Complex reviews, Mapping, Methodology, Mixed methods reviews, Research methods, Scoping reviews, Synthesis, Systematic reviews, Taxonomy of reviews

## Abstract

This paper argues that the current proliferation of types of systematic reviews creates challenges for the terminology for describing such reviews. Terminology is necessary for planning, describing, appraising, and using reviews, building infrastructure to enable the conduct and use of reviews, and for further developing review methodology. There is insufficient consensus on terminology for a typology of reviews to be produced and any such attempt is likely to be limited by the overlapping nature of the dimensions along which reviews vary. It is therefore proposed that the most useful strategy for the field is to develop terminology for the main dimensions of variation. Three such main dimensions are proposed: (1) aims and approaches (including what the review is aiming to achieve, the theoretical and ideological assumptions, and the use of theory and logics of aggregation and configuration in synthesis); (2) structure and components (including the number and type of mapping and synthesis components and how they relate); and (3) breadth and depth and the extent of ‘work done’ in addressing a research issue (including the breadth of review questions, the detail with which they are addressed, and the amount the review progresses a research agenda). This then provides an overarching strategy to encompass more detailed descriptions of methodology and may lead in time to a more overarching system of terminology for systematic reviews.

## Background

Research studies vary in many ways including the types of research questions they are asking, the reasons these questions are being asked, the theoretical and ideological perspectives underlying these questions, and in the research methods that they employ. Systematic reviews are a form of research; they are (and the theoretical and ideological perspectives underlying these methods) a way of bringing together what is known from the research literature using explicit and accountable methods [1]. Systematic methods of review have been successfully developed particularly for questions concerning the impact of interventions; these synthesize the findings of studies which use experimental controlled designs. Yet the logic of systematic methods for reviewing the literature can be applied to all areas of research; therefore there can be as much variation in systematic reviews as is found in primary research [2,3]. This paper discusses some of the important conceptual and practical differences between different types of systematic review. It does not aim to provide an overall taxonomy of all types of reviews; the rate of development of new approaches to reviewing is too fast and the overlap of approaches too great for that to be helpful. Instead, the paper argues that, for the present at least, it is more useful to identify the key dimensions on which reviews differ and to examine the multitude of different combinations of those dimensions. The paper also does not aim to describe all of the myriad actual and potential differences between reviews; this would be a task too large even for a book let alone a paper. The focus instead is on three major types of dimensions of difference. The first dimension is the aims and approaches of reviews; particularly in terms of their methodologies (their ontological and epistemological foundations and methods of synthesis). The second dimension is the structure and components of reviews. The third dimension is the breadth, depth, and extent of the work done by a review in engaging with a research issue. Once these three aspects of a review are clear, consideration can be given to more specific methodological issues such as methods of searching, identifying, coding, appraising, and synthesizing evidence. The aim of this paper is to clarify some of the major conceptual distinctions between reviews to assist the selection, evaluation, and development of methods for reviewing.

### Clarifying the nature of variation in reviews

As forms of research, systematic reviews are undertaken according to explicit methods. The term ‘systematic’ distinguishes them from reviews undertaken without clear and accountable methods.

The history of systematic reviews is relatively recent [4,5] and despite early work on meta-ethnography [6], the field has been dominated by the development and application of statistical meta-analysis of controlled trials to synthesize the evidence on the effectiveness of health and social interventions. Over the past 10 years, other methods for reviewing have been developed. Some of these methods aim to extend effectiveness reviews with information from qualitative studies [7]. The qualitative information may be used to inform decisions made in the statistical synthesis or be part of a mixed methods synthesis (discussed later). Other approaches have been developed from a perspective which, instead of the statistical aggregation of data from controlled trials, emphasize the central role that theory can play in synthesizing existing research [8,9], address the complexity of interventions [10], and the importance of understanding research within its social and paradigmatic context [11]. The growth in methods has not been accompanied by a clear typology of reviews. The result is a complex web of terminology [2,12].

The lack of clarity about the range of methods of review has consequences which can limit their development and subsequent use. Knowledge or consensus about the details of specific methods may be lacking, creating the danger of the over-generalization or inappropriate application of the terminology being used. Also, the branding of different types of review can lead to over-generalizations and simplification with assumptions being made about differences between reviews that only apply to particular stages of a review or that are matters of degree rather than absolute differences. For example, concepts of quality assurance can differ depending upon the nature of the research question being asked. Similarly, infrastructure systems developed to enable the better reporting and critical appraisal of reviews, such as PRISMA [13], and for registration of reviews, such as PROSPERO [14] currently apply predominantly to a subset of reviews, the defining criteria of which may not be fully clear.

A further problem is that systematic reviews have attracted criticism on the assumption that systematic reviewing is applicable only to empirical quantitative research [15]. In this way, polarized debates about the utility and relevance of different research paradigms may further complicate terminological issues and conceptual understandings about how reviews actually differ from one another. All of these difficulties are heightened because review methods are undergoing a period of rapid development and so the methods being described are often being updated and refined.

Knowledge about the nature and strengths of different forms of review is necessary for: appropriate choice of review methods by those undertaking reviews; consideration of the importance of different issues of quality and relevance for each stage of a review; appropriate and accurate reporting and accountability of such review methods; interpretation of reviews; commissioning of reviews; development of procedures for assessing and undertaking reviews; and development of new methods.

Clarifying the nature of the similarities and differences between reviews is a first step to avoiding these potential limitations. A typology of review methods might be a solution. There are many diverse approaches to reviews that can be easily distinguished, such as statistical meta-analysis and meta-ethnography. A more detailed examination, however, reveals that the types of review currently described often have commonalities that vary across types of review and at different stages of a review. Three of these dimensions are described here. Exploring these dimensions also reveals how reviews differ in degree along these overlapping dimensions rather than falling into clear categories.

## Review aims and approaches

Primary research and research reviews vary in their ontological, epistemological, ideological, and theoretical stance, their research paradigm, and the issues that they aim to address. In reviews, this variation occurs in both the method of review and the type of primary research that they consider. As reviews will include primary studies that address the focus of the review question, it is not surprising that review methods also tend to reflect many of the approaches, assumptions, and methodological challenges of the primary research that they include.

One indication of the aim and approach of a study is the research question which the study aims to answer. Questions commonly addressed by systematic reviews include: what is the effect of this intervention (addressed by, for example, the statistical meta-analysis of experimental trials); what is the accuracy of this diagnostic tool (addressed by, for example, meta-analysis of evaluations of diagnostic tests); what is the cost of this intervention (addressed by, for example, a synthesis of cost-benefit analyses); what is the meaning or process of a phenomena (addressed by, for example, conceptual synthesis such as meta-ethnography or a critical interpretative synthesis of ethnographic studies); what is the effect of this complex intervention (addressed by, for example, multi-component mixed methods reviews); what is the effect of this approach to social policy in this context (addressed by, for example, realist synthesis of evidence of efficacy and relevance across different policy areas); and what are the attributes of this intervention or activity (addressed by, for example, framework synthesis framed by dimensions explicitly linked to particular perspectives).

Although different questions drive the review process and suggest different methods for reviewing (and methods of studies included) there is considerable overlap in the review methods that people may select to answer these questions; thus the review question alone does not provide a complete basis for generating a typology of review methods.

### Role of theory

There is no agreed typology of research questions in the health and social sciences. In the absence of such a typology, one way to distinguish research is in the extent that it is concerned with generating, exploring, or testing theory [16].

In addressing an impact question using statistical meta-analysis, the approach is predominantly the empirical testing of a theory that the intervention works. The theory being tested may be based on a detailed theory of change (logic model) or be a ‘black box’ where the mechanisms by which change may be affected are not articulated. The review may, in addition to testing theory, include methods to generate hypotheses about causal relations. Testing often (though not always) wants to add up or aggregate data from large representative samples to obtain a more precise estimate of effect. In the context of such reviews, searching aims to identify a representative sample of studies, usually by attempting to include all relevant studies in order to avoid bias from study selection (sometimes called ‘exhaustive’ searching). Theoretical work in such analyses is undertaken predominantly before and after the review, not during the review, and is concerned with developing the hypothesis and interpreting the findings.

In research examining processes or meanings the approach is predominantly about developing or exploring theory. This may not require representative samples of studies (as in aggregative reviews) but does require variation to enable new conceptual understandings to be generated. Searching for studies in these reviews adopts a theoretical approach to searching to identify a sufficient and appropriate range of studies either through a rolling sampling of studies according to a framework that is developed inductively from the emerging literature (akin to theoretical sampling in primary research) [17]; or through a sampling framework based on an existing body of literature (akin to purposive sampling in primary research) [18]. In both primary research and reviews, theoretical work is undertaken during the process of the research; and, just as with the theory testing reviews, the nature of the concepts may be relatively simple or very complex.

### Aggregative and configurative reviews

**Figure 1:**
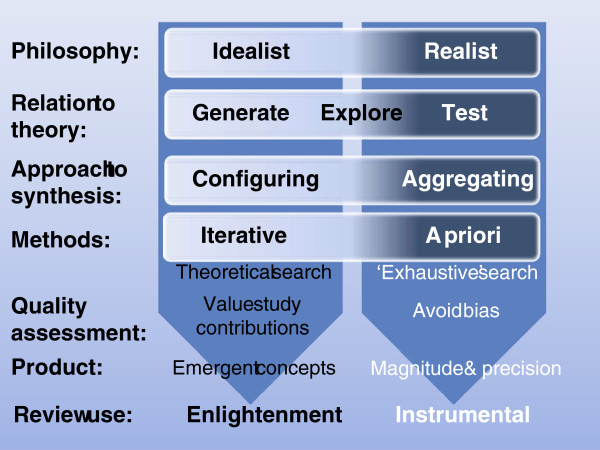
Continua of approaches in aggregative and configurative reviews.

The distinction between research that tests and research that generates theory also equates to the distinction between review types made by Voils, Sandelowski and colleagues [19,20] (although we have been very influenced by these authors the detail of our use of these terms may differ in places). Reviews that are collecting empirical data to describe and test predefined concepts can be thought of as using an ‘aggregative’ logic. The primary research and reviews are adding up (aggregating) and averaging empirical observations to make empirical statements (within predefined conceptual positions). In contrast, reviews that are trying to interpret and understand the world are interpreting and arranging (configuring) information and are developing concepts (Figure 1). This heuristic also maps onto the way that the review is intended to inform knowledge. Aggregative research tends to be about seeking evidence to inform decisions whilst configuring research is seeking concepts to provide enlightenment through new ways of understanding. 

Aggregative reviews are often concerned with using predefined concepts and then testing these using predefined (*a priori*) methods. Configuring reviews can be more exploratory and, although the basic methodology is determined (or at least assumed) in advance, specific methods are sometimes adapted and selected (iteratively) as the research proceeds. Aggregative reviews are likely to be combining similar forms of data and so be interested in the homogeneity of studies. Configurative reviews are more likely to be interested in identifying patterns provided by heterogeneity [12].

The logic of aggregation relies on identifying studies that support one another and so give the reviewer greater certainty about the magnitude and variance of the phenomenon under investigation. As already discussed in the previous section, the approach to searching for studies to include (the search strategy) is attempting to be exhaustive or, if not exhaustive, then at least avoiding bias in the way that studies are found. Configuring reviews have the different purpose of aiming to find sufficient cases to explore patterns and so are not necessarily attempting to be exhaustive in their searching. (Most reviews contain elements of both aggregation and configuration and so some may require an unbiased set of studies as well as sufficient heterogeneity to permit the exploration of differences between them).

Aggregating and configuring reviews also vary in their approach to quality assurance. All reviews aim to avoid drawing misleading conclusions because of problems in the studies they contain. Aggregative reviews are concerned with *a priori* methods and their quality assurance processes assess compliance with those methods. As the basis of quality assurance is known *a priori*, many aspects of this can be incorporated into the inclusion criteria of the review and then can be further checked at a later quality assurance stage. The inclusion criteria may, for example, require only certain types of study with specific methodological features. There is less consensus in the practice of quality assessment in configurative reviews; some adopt a similar strategy to those employed in aggregative reviews, whereas others reject the idea that the quality of a study can be assessed through an examination of its method, and instead prioritize other issues, such as relevance to the review and the contribution the study can make in the review synthesis to testing or generating theory [21-23]. Some of the differences between aggregating and configuring reviews are shown in Figure 1.

Although the logics of aggregating and configuring research findings demand different methods for reviewing, a review often includes components of both. A meta-analysis may contain a *post hoc* interpretation of statistical associations which may be configured to generate hypotheses for future testing. A configurative synthesis may include some components where data are aggregated (for example, framework synthesis) [24,25]. Examples of reviews that are predominantly aggregative, configurative, or with high degrees of both aggregation and configuring are given in Table 1 (and for a slightly different take on this heuristic see Sandelowski *et al.*[20]). 

**Table 1:** Examples of review types

**Predominant review type**	**Review questions**
*Aggregative*	
‘What works?’ reviews	What is the effect of a health or social intervention?
Diagnostic test	What is the accuracy of this diagnostic tool?
Cost benefit	How effective is the benefit of an intervention relative to its cost?
Prevalence	How extensive is this condition?
*Configurative*	
Meta-ethnography [4]	What theories can be generated from the conceptual literature?
Critical interpretative synthesis [8]	What theories can be generated from the conceptual literature?
Meta narrative review [11]	How to understand the development of research on an issue within and across different research traditions?
*Configuring and aggregative*	
Realist synthesis [9]	What is the effect of a social policy in different policy areas?
Framework synthesis [25]	What are the attributes of an intervention or activity?

Similarly, the nature of a review question, the assumptions underlying the question (or conceptual framework), and whether the review aggregates or configures the results of other studies may strongly suggest which methods of review are appropriate, but this is not always the case. Several methods of review are applicable to a wide range of review approaches. Both thematic [26] and framework synthesis [24,25] which identify themes within narrative data can, for example, be used with both aggregative and configurative approaches to synthesis.

Reviews that are predominantly aggregative may have similar epistemological and methodological assumptions to much quantitative research and there may be similar assumptions between predominantly configurative reviews and qualitative research. However, the quantitative/qualitative distinction is not precise and does not reflect the differences in the aggregative and configurative research processes; quantitative reviews may use configurative processes and qualitative reviews can use aggregative processes. Some authors also use the terms conceptual synthesis for reviews that are predominantly configurative, but the process of configuring in a review does not have to be limited to concepts; it can also be the arrangement of numbers (as in subgroup analyses of statistical meta-analysis). The term ‘interpretative synthesis’ is also used to describe reviews where meanings are interpreted from the included studies. However, aggregative reviews also include interpretation, before inspection of the studies to develop criteria for including studies, and after synthesis of the findings to develop implications for policy, practice, and further research. Thus, the aggregate/configure framework cannot be thought of as another way of expressing the qualitative/quantitative ‘divide’; it has a more specific meaning concerning the logic of synthesis, and many reviews have elements of both aggregation and configuration.

### Further ideological and theoretical assumptions

In addition to the above is a range of issues about whose questions are being asked and the implicit ideological and theoretical assumptions driving both them and the review itself. These assumptions determine the specific choices made in operationalizing the review question and thus determine the manner in which the review is undertaken, including the research studies included and how they are analyzed. Ensuring that these assumptions are transparent is therefore important both for the execution of the review and for accountability. Reviews may be undertaken to inform decision-making by non-academic users of research such as policymakers, practitioners, and other members of the public and so there may be a wide range of different perspectives that can inform a review [27,28]. The perspectives driving the review will also influence the findings of the review and thereby clarify what is known and not known (within those perspectives) and thus inform what further primary research is required. Both reviewer and user perspectives can thus have an ongoing influence in developing user-led research agendas. There may be many different agendas and thus a plurality of both primary research and reviews of research on any given issue.

A further fundamental issue that is related to the types of questions being asked and the ideological and theoretical assumptions underlying them is the ontological and epistemological position taken by the reviewers. Aggregative reviews tend to assume that there is (often within disciplinary specifications/boundaries) a reality about which empirical statements can be made even if this reality is socially constructed (generalizations); in other words they take a ‘realist’ philosophical position (a broader concept than the specific method of ‘realist synthesis’). Some configurative reviews may not require such realist assumptions. They take a more relativist idealist position; the interest is not in seeking a single ‘correct’ answer but in examining the variation and complexity of different conceptualizations [12,29]. These philosophical differences can be important in understanding the approach taken by different reviewers just as they are in understanding variation in approach (and debates about research methods) in primary research. These differences also relate to how reviews are used. Aggregative reviews are often used to make empirical statements (within agreed conceptual perspectives) to inform decision making instrumentally whilst configuring reviews are often used to develop concepts and enlightenment [30].

## Structure and components of reviews

As well as varying in their questions, aims, and philosophical approach, reviews also vary in their structure. They can be single reviews that synthesize a specific literature to answer the review question. They may be maps of what research has been undertaken that are products in their own right and also a stage on the way to one or more syntheses. Reviews can also contain multiple components equating to conducting many reviews or to reviewing many reviews.

### Systematic maps

**Figure 2:**
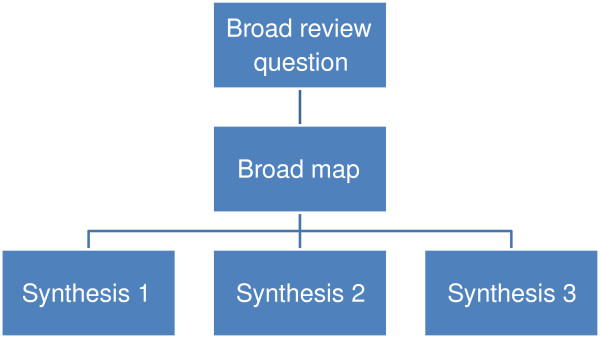
A map leading to several syntheses.

**Figure 3:**
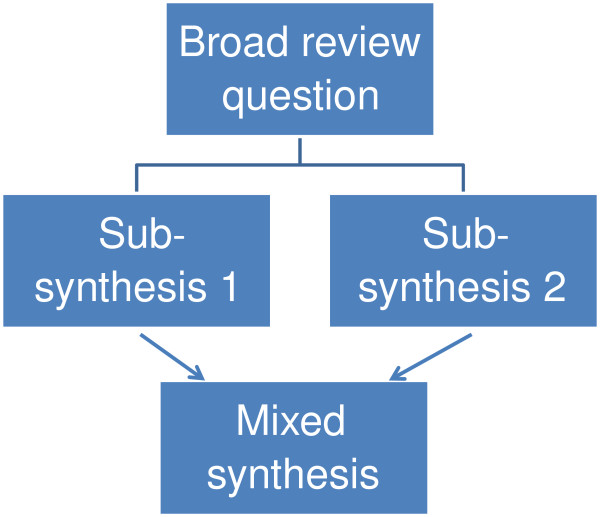
A mixed method review with three syntheses.

To some degree, most reviews describe the studies they contain and thus provide a map or account of the research field. Some reviews go further than this and more explicitly identify aspects of the studies that help describe the research field in some detail; the focus and extent of such description varying with the aims of the map. Maps are useful products in their own right but can also be used to inform the process of synthesis and the interpretation of the synthesis [3,30]. Instead of automatically undertaking a synthesis of all included studies, an analysis of the map may lead to a decision to synthesize only a subset of studies, or to conduct several syntheses in different areas of the one map. A broader initial review question and a narrower subsequent review question allows the synthesis of a narrower subset of studies to be understood within the wider literature described in terms of research topics, primary research methods, or both. It also allows broader review questions to create a map for a series of reviews (Figure 2) or mixed methods reviews (Figure 3). In sum, maps have three main purposes of: (i) describing the nature of a research field; (ii) to inform the conduct of a synthesis; and (iii) to interpret the findings of a synthesis [3,31].The term ‘scoping review’ is also sometimes used in a number of different ways to describe (often non-systematic) maps and/or syntheses that rapidly examine the nature of the literature on a topic area [32,33]; sometimes as part of the planning for a systematic review. 

### Mixed methods reviews

The inclusion criteria of a review may allow all types of primary research or only studies with specific methods that are considered most appropriate to best address the review question. Including several different methods of primary research in a review can create challenges in the synthesis stage. For example, a review asking about the impact of some life experience may examine both randomized controlled trials and large data sets on naturally occurring phenomena (such as in large scale cohort studies). Another strategy is to have sub-reviews that ask questions about different aspects of an issue and which are likely to consider different primary research [34,35]. For example, a statistical meta-analysis of impact studies compared with a conceptual synthesis of people’s views of the issue being evaluated [34,35]. The two sub-reviews can then be combined and contrasted in a third synthesis as in Figure 3. Mixed methods reviews have many similarities with mixed methods in primary research and there are therefore numerous ways in which the products of different synthesis methods may be combined [35].

**Figure 4:**
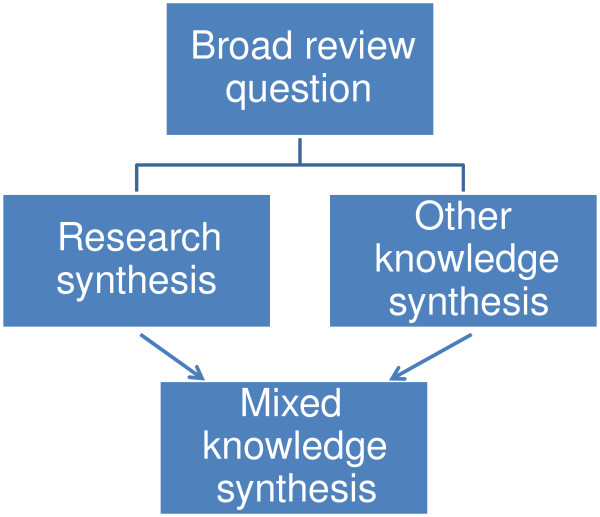
Mixed knowledge review.

Mixed knowledge reviews use a similar approach but combine data from previous research with other forms of data; for example a survey of practice knowledge about an issue (Figure 4).

Another example of a mixed methods review is realist synthesis [9] that examines the usefulness of mid-level policy interventions across different areas of social policy by unpacking the implicit models of change, followed by an iterative process of identifying and analyzing the evidence in support of each part of that model. This is quite similar to a theory-driven aggregative review (or series of reviews) that aggregatively test different parts of a causal model. The first part of the process is a form of configuration in clarifying the nature of the theory and what needs to be empirically tested; the second part is the aggregative testing of those subcomponents of the theory. The difference between this method and more ‘standard’ systematic review methods is that the search for empirical evidence is more of an iterative, investigative process of tracking down and interpreting evidence. Realist synthesis will also consider a broad range of empirical evidence and will assess its value in terms of its contribution rather than according to some preset criteria. The approach therefore differs from the predominantly *a priori* strategy used in either standard ‘black box’ or in theory driven aggregative reviews. There have also been attempts to combine aggregative ‘what works’ reviews with realist reviews [36]. These innovations are exploring how best to develop the breadth, generalizability and policy relevance of aggregative reviews without losing their methodological protection against bias.

There are also reviews that use other pre-existing reviews as their source of data. These reviews of reviews may draw on the data of previous reviews either by using the findings of previous reviews or by drilling down to using data from the primary studies in the reviews [37]. Information drawn from many reviews can also be mined to understand more about a research field or research methods in meta-epidemiology [38]. As reviews of reviews and meta-epidemiology both use reviews as their data, they are sometimes both described as types of ‘meta reviews’. This terminology may not be helpful as it links together two approaches to reviews which have little in common apart from the shared type of data source. A further term is ‘meta evaluation’. This can refer to the formative or summative evaluation of primary evaluation studies or can be a summative statement of the findings of evaluations which is a form of aggregative review (See Gough *et al.* in preparation, and [39]).

## Breadth, depth, and ’work done’ by reviews

Primary research studies and reviews may be read as isolated products yet they are usually one step in larger or longer-term research enterprises. A research study usually addresses a macro research issue and a specific focused sub-issue that is addressed by its specific data and analysis [16]. This specific focus can be broad or narrow in scope and deep or not so deep in the detail in which it is examined.

### Breadth of question

Many single component aggregative reviews aim for homogeneity in the focus and method of included studies. They select narrowly defined review questions to ensure a narrow methodological focus of research findings. Although well justified, these decisions may lead to each review providing a very narrow view of both research and the issue that is being addressed. A user of such reviews may need to take account of multiple narrow reviews in order to help them determine the most appropriate course of action.

The need for a broader view is raised by complex questions. One example is assessing the impact of complex interventions. There are often many variants of an intervention, but even within one particular highly specified intervention there may be variations in terms of the frequency, duration, degree, engagement, and fidelity of delivery [40]. All of this variation may result in different effects on different participants in different contexts. The variation may also impact differentially within the hypothesized program theory of how the intervention impacts on different causal pathways. Reviews therefore need a strategy for how they can engage with this complexity. One strategy is to achieve breadth through multi-component reviews; for example, a broad map which can provide the context for interpreting a narrower synthesis, a series of related reviews, or mixed methods reviews. Other strategies include ‘mega reviews’, where the results from very many primary studies or meta-analyses are aggregated statistically (for example, [41,42]) and multivariate analyses, where moderator variables are used to identify the ‘active ingredients’ of interventions (for example, [43,44]). Whether breadth is achieved within a single review, from a sequence of reviews, from reviews of reviews, or from relating to the primary and review work of others, the cycle of primary research production and synthesis is part of a wider circle of engagement and response to users of research [45].

### Review resources and breadth and depth of review

The resources required for a systematic review are not fixed. With different amounts of resource one can achieve different types of review. Broad reviews such as mixed methods and other multi-component reviews are likely to require more resources, all else being constant, than narrow single method reviews. Thus, in addition to the breadth of review is the issue of its depth, or the detail with which it is undertaken. A broad review may not have greater resources than a narrow review in which case those resources are spread more thinly and each aspect of that breadth may be undertaken with less depth.

When time and other resources are very restricted then a rapid review may be undertaken where some aspect of the review will be limited; for example, breadth of review question, sources searched, data coded, quality and relevance assurance measures, and depth of analysis [46,47]. Many students, for example, undertake literature reviews that may be informed by systematic review principles of rigor and transparency of reporting; some of these maybe relatively modest exercises whilst others make up a substantial component of the thesis. If rigor of execution and reporting are reduced too far then it may be more appropriate to characterize the work as non systematic scoping than as a systematic review.

Reviews thus vary in the extent that they engage with a research issue. The enterprise may range in size from, for instance, a specific program theory to a whole field of research. The enterprise may be under study by one research team, by a broader group such as a review group in an international collaboration or be the focus of study by many researchers internationally. The enterprises may be led academic disciplines, applied review collaborations, by priority setting agendas, and by forums to enable different perspectives to be engaged in research agendas. Whatever the nature of the strategic content or process of these macro research issues, reviews vary in the extent that they plan to contribute to such more macro questions. Reviews thus vary in the extent that this research work is done within a review; rather than before and after a review (by primary studies or by other reviews).

Reviews can be undertaken with different levels of skill, efficiency, and automated tools [48] and so resources do not equate exactly with the ‘work done’ in progressing a research issue. In general, a broad review with relatively little depth (providing a systematic overview) may be comparable in work done to a detailed narrow review (as in many current statistical meta-analyses). A multi-component review addressing complex questions using both aggregative and configuring methods may be attempting to achieve more work, though there may be challenges in terms of maintaining consistency or transparency of detail in each component of the review. In contrast, a rapid review has few resources and so is attempting less than other reviews but there may be dangers that the limited scope (and limited contribution to the broader research agenda) is not understood by funders and users of the review. How best to use available resources is a strategic issue depending upon the nature of the review question, the state of the research available on that issue and the knowledge about that state of the research. It is an issue of being fit for purpose. A review doing comparatively little ‘work’ may be exactly what is needed in one situation but not in another.

## Conclusion

Explicit accountable methods are required for primary research and reviews of research. This logic applies to all research questions and thus multiple methods for reviews of research are required, just as they are required for primary research. These differences in types of reviews reflect the richness of primary research not only in the range of variation but also in the philosophical and methodological challenges that they pose including the mixing of different types of methods. The dominance of one form of review question and review method and the branding of some other forms of review does not clearly describe the variation in review designs and methods and the similarities and differences between these methods. Clarity about the dimensions along which reviews vary provides a way to develop review methods further and to make critical judgments necessary for the commission, production, evaluation, and use of reviews. This paper has argued for the need for clarity in describing the design and methods of systematic reviews along many dimensions; and that particularly useful dimensions for planning, describing, and evaluating reviews are:

1. Review aims and approach: (i) approach of the review: ontological, epistemological, theoretical, and ideological assumptions of the reviewers and users of the review including any theoretical mode; (ii) review question: the type of answer that is being sought (and the type of information that would answer it); and (ii) aggregation and configuration: the relative use of these logics and strategies in the different review components (and the positioning of theory in the review process, the degree of homogeneity of data, and the iteration of review method).

2. Structure and components of reviews: (iv) the systematic map and synthesis components of the review; and (v) the relation between these components.

3. Breadth, depth, and ‘work done’ by reviews: (vi) macro research strategy: the positioning of the review (and resources and the work aimed to be done) within the state of what is already known and other research planned by the review team and others; and (vii) the resources used to achieve this.

Clarifying some of the main dimensions along which reviews vary can provide a framework within which description of more detailed aspects of methodology can occur; for example, the specific strategies used for searching, identifying, coding, and synthesizing evidence and the use of specific methods and techniques ranging from review management software to text mining to statistical and narrative methods of analysis. Such clearer descriptions may lead in time to a more overarching system of terminology for systematic reviews.

## Competing interests

The authors declare that they have no competing interests.

## **Authors’ contributions**

All three authors have made substantial contributions to the conception of the ideas in this paper, have been involved in drafting or revising it critically for important intellectual content, and have given final approval of the version to be published.

## **Authors’ information**

DG, JT, and SO are all directors of the Evidence for Policy and Practice Information and Coordinating Centre (EPPI-Centre) [49].
